# β cell membrane remodelling and procoagulant events occur in inflammation‐driven insulin impairment: a GLP‐1 receptor dependent and independent control

**DOI:** 10.1111/jcmm.12683

**Published:** 2015-11-26

**Authors:** Céline Gleizes, Guillaume Kreutter, Malak Abbas, Mohamad Kassem, Andrei Alexandru Constantinescu, Julie Boisramé‐Helms, Blandine Yver, Florence Toti, Laurence Kessler

**Affiliations:** ^1^ EA7293, Vascular and Tissular Stress in TransplantationFaculty of MedicineUniversity of StrasbourgIllkirchFrance; ^2^ Doctoral School of Sciences and TechnologiesLebanese UniversityBeiruth‐HadathLebanon; ^3^ Department of Parasitology and Parasitic Diseases and Animal BiologyFaculty of Veterinary MedicineUniversity of Agronomical Sciences and Veterinary MedicineBucharestRomania; ^4^ Department of ReanimationNouvel hopital civilStrasbourg CEDEXFrance; ^5^ Federation of Translational Medicine of StrasbourgFaculty of MedicineUniversity of StrasbourgStrasbourgFrance; ^6^ UMR7213 CNRSLaboratory of Biophotonics and PharmacologyFaculty of PharmacyUniversity of StrasbourgIllkirchFrance; ^7^ Department of DiabetologyUniversity HospitalStrasbourg CedexFrance

**Keywords:** insulin, β cell, microparticles, tissue factor, lipid raft, exocytosis, ion channels, GLP‐1 receptor

## Abstract

Inflammation and hyperglycaemia are associated with a prothrombotic state. Cell‐derived microparticles (MPs) are the conveyors of active procoagulant tissue factor (TF) and circulate at high concentration in diabetic patients. Liraglutide, a glucagon‐like peptide (GLP)‐1 analogue, is known to promote insulin secretion and β‐cell preservation. In this *in vitro* study, we examined the link between insulin impairment, procoagulant activity and plasma membrane remodelling, under inflammatory conditions. Rin‐m5f β‐cell function, TF activity mediated by MPs and their modulation by 1 μM liraglutide were examined in a cell cross‐talk model. Methyl‐β‐cyclodextrine (MCD), a cholesterol depletor, was used to evaluate the involvement of raft on TF activity, MP shedding and insulin secretion as well as Soluble N‐éthylmaleimide‐sensitive‐factor Attachment protein Receptor (SNARE)‐dependent exocytosis. Cytokines induced a two‐fold increase in TF activity at MP surface that was counteracted by liraglutide. Microparticles prompted TF activity on the target cells and a two‐fold decrease in insulin secretion *via* protein kinase A (PKA) and p38 signalling, that was also abolished by liraglutide. Large lipid raft clusters were formed in response to cytokines and liraglutide or MCD‐treated cells showed similar patterns. Cells pre‐treated by saturating concentration of the GLP‐1r antagonist exendin (9‐39), showed a partial abolishment of the liraglutide‐driven insulin secretion and liraglutide‐decreased TF activity. Measurement of caspase 3 cleavage and MP shedding confirmed the contribution of GLP‐1r‐dependent and ‐independent pathways. Our results confirm an integrative β‐cell response to GLP‐1 that targets receptor‐mediated signalling and membrane remodelling pointing at the coupling of insulin secretion and inflammation‐driven procoagulant events.

## Introduction

In diabetes patients, MPs, that are surrogates of cell activation, were reported to circulate at high concentration, even in well‐controlled type 2 diabetes (T2DM) patients 1, 2, 3, 4. Microparticles are submicron fragments of the plasma membrane released in biological fluids and in the peri‐cellular environment under conditions of metabolic or apoptotic stress 5, 6. The release of MPs is prompted by a drastic plasma membrane remodelling and the translocation of anionic phospholipids from the inner to the outer leaflet. Microparticles contain a broad array of imbedded active proteins and therefore act as cellular effectors through the delivery of biological signals to target cells. In the vessel, MPs support coagulation owing to the exposure of the anionic phospholipid phosphatidylserine (PhSer) and to the presence of active TF 7, 8. Tissue factor is the membrane initiator of coagulation and controlled by an early responsive gene, the expression of which is induced under pro‐inflammatory conditions, mostly in endothelial and monocyte cells 9. Highly procoagulant MPs of endothelial origin and conveying active TF are detected in patients with diabetes 10, and were associated with prothrombotic state 11, 12, 13. In stimulated cells, TF activity at cell surface is potentiated by the exposed PhSer. Lipid rafts are dynamic cholesterol‐enriched microdomains that contribute to TF activity and its regulation by ensuring the spatial clustering of TF and exposed PhSer 14, 15, 16. Relationships between lipid rafts and insulin secretion have been reported in studies 17, 18, 19, 20, describing the regulation of ion channels and exocytosis, particularly *via* raft‐embedded SNARE proteins 21, 22.

Liraglutide is a GLP‐1 analogue that belongs to the incretinomimetics class of drugs. In the treatment of T2DM, the beneficial effects of liraglutide rely on their ability to improve glycemic control, insulin secretion and promote β‐cell survival 23, 24, 25. In a previous work, we have shown that Liraglutide decreases TF activity measured at β‐cell surface and reduces MPs shedding under oxidative and cytokine stress conditions 26.

In the present work, we investigated the role of TF‐bearing MPs on the impairment of insulin secretion by Rin‐m5f β cells, submitted to prolonged hyperglycaemic conditions and pro‐inflammatory stress. Because MP shedding is the consequence of membrane remodelling and TF activity is potentiated by PhSer translocation across the membrane as well as raft concentration, we investigated the effect of liraglutide and raft disruption on TF activity and insulin secretion. The incidence of the GLP‐1 receptor (GLP‐1r) signalling was investigated using exendin (9‐39), a GLP‐1r antagonist.

## Materials and methods

### Cell culture

Rat β cells, Rin‐m5f (CRL‐11605^™^; ATCC, Manassas, VA, USA), were seeded at 125,000 cells/cm^2^ in RPMI 1640 medium (PAN^™^ Biotech GmbH, Aidenbach, Germany) containing 4.5% glucose, 10 mM HEPES, (4‐(2‐hydroxyethyl)‐1‐piperazineethanesulfonic acid) 2 mM glutamine, 1 mM sodium pyruvate and supplemented with 10% foetal bovine serum (Gibco, Saint Aubin, France) and 20 μg/ml gentamycine (Lonza, Basel, Switzerland). Cells were cultured at 37°C and 5% CO_2_ in a humidified atmosphere.

### Cellular models of stress and pharmacological modulation

Rin‐m5f were chosen as an adequate model for the study of the β‐cell response to prolonged inflammation and hyperglycaemia, submitted to 24–48 hrs cytokine and oxidative stress. Indeed Rin‐m5f are not responsive to a short metabolic raise by glucose stimulation, but develop apoptosis after prolonged exposure to H_2_O_2_ 26. Stress was applied when cells reached 70% of confluence as reported elsewhere 27. Inflammatory stress was induced by a 24 hrs treatment with the combination of 50 U/ml of IL‐1β (Sigma‐Aldrich, St. Louis, MO, USA) and 1000 U/ml of TNF‐α (Sigma‐Aldrich), further referred to as ‘cytokines’ throughout the manuscript. Cytokine effects were compared to those prompted by H_2_O_2_ application, a well‐established treatment leading to Rin‐m5f dysfunction. Oxidative stress was induced by 100 μM H_2_O_2_ in fresh medium during 6 hrs. Cell supernatants were collected at the end of each stress procedure and kept at 4°C until measurement.

Pharmacological inhibition of PKA was achieved by pre‐treatment with 10 μM H89 during 30 min. before 24 hrs incubation with MPs. Inhibition of K^+^‐ATP channels and Ca^2+^ channels was performed by continuous exposure to 10 μM Amlodipine and 0.25 mM Diazoxide, for the cytokine or H_2_O_2_ respective incubation times. In all experiments, liraglutide (Novo Nodisk, Bagsvaerd, Denmark) was added at the concentration of 1 μM as proposed by other investigators 28, 29, 30, 31.

### Insulin measurement

Insulin released in the supernatant after 24 hrs, was assessed by ELISA assay with the matrix solution, according to supplier recommendations (ELISA Kit Rat/Mouse Insulin; Millipore, Molsheim, France).

### MP generation, harvest, and quantification

Microparticles were harvested from the supernatants of stimulated cells under sterile conditions 24 hrs after the initiation of the cytokine or H_2_O_2_ treatment (see above and as described elsewhere 26). Detached cells and debris were discarded by differential centrifugation steps and MPs washed in HBSS and concentrated by two‐centrifugation steps (13,000 × g, 1 hr) and kept at 4°C for not more than 3 weeks.

Total MP concentration was determined by prothrombinase assay as previously described 26. Briefly, MP captured onto insolubilized Annexin‐5 were incubated with blood clotting factors (FXa, FVa, FII) and CaCl_2_ 32. Conversion of prothromobin to thrombin was revealed by chromogenic substrate, using a spectrophotometric reader at 405 nm. Results were expressed as nanomolar PhtdSer equivalent (nM PhtdSer eq.) by reference to a standard curve constructed using liposomes of known concentration and PhtdSer eq. proportion 33.

### MP‐mediated cell cross‐talk

Microparticles generated by oxidative stress (MPox) and by cytokine stress (MPcyt) were applied to naïve Rin‐m5f cells (70% confluence) at a final concentration of 10 nM PhtdSer eq. during 24 hrs. In some experiments, 1 μM liraglutide was added to the cell medium and isolated MPs could be pre‐incubated with an antibody to tissue factor (HTF‐1, kind gift of Prof. N. Mackmann, Chapel Hill, USA).

### Measurement of TF activity

After 6 hrs stimulation, TF activity was measured in supernatants and at the surface of washed target cells through its ability to promote the activation of factor X (150 nM; Hyphen Biomed, Neuville‐sur‐Oise, France) by factor VII(a) (5 nM; Novoseven, Hillerød, Denmark). The reaction was allowed to proceed for 15 min. at 37°C, 0.1 mM CS11, a chromogenic substrate for factor Xa (Hyphen Biomed, Neuville‐sur‐Oise, France), were added and absorbance recorded at 405 nm (65). Results were expressed as fM TF activity per 50,000 living cells by reference to a standard curve established with known amounts of highly purified, lipidated recombinant human tissue factor (ADF Biomedical, Neuville‐sur‐Oise, France).

### Western blot analysis

After treatment, cells were washed twice with PBS and then lysed in TRIS (trishydroxyméthylaminométhane) buffer containing protease inhibitors (5 μg/ml leupeptin, 5 mM benzamidine) and 2% Triton^®^ X‐100 on ice. Total proteins (30 μg) were separated by electrophoresis on 10% SDS‐polyacrylamide (Sigma‐Aldrich) gels as previously described 34. Blotting membranes were incubated with the different primary antibodies directed against rat‐phosphorylated p38 (1:1000 dilution; Santa Cruz Biotechnology, Santa Cruz, CA, USA), rat‐cleaved caspase 3 (1:1000 dilution; Cell Signaling Technology, Danvers, MA, USA) and rat GLP‐1 receptor (1:1000 dilution; Alomone Labs, Jerusalem, Israel), overnight at 4°C. Detection of β‐tubulin was used for normalization. After washing, membranes were incubated with the secondary antimouse IgG antibody (1:10,000 dilution; Cell Signaling Technology) at room temperature for 60 min. Pre‐stained markers (Invitrogen^™^, Carlsbad, CA, USA) were used for molecular mass determinations. Immunoreactive bands were detected by enhanced chemiluminescence (GE Healthcare, Amersham, UK). Density analysis was performed with ImageQuant LAS 4000 imager (GE Healthcare).

### TF labelling

Cells were submitted to both stress for1 hr up to 8 hrs, washed, fixed with Fix and Perm^®^ (Sigma‐Aldrich), and kept at 4°C before incubation with FITC‐conjugated (Fluorescein isothiocyanate) rabbit anti‐rat TF (dilution: 1:50; Life Science), Saint Louis, MO, USA during 30 min. in darkness. Tissue factor expression‐associated green fluorescence was quantified by flow cytometry (FACS‐scan cytometer; Becton Dickinson, San José, CA, USA) set at logarithmic gain. Around 10,000 events were recorded for each sample.

### Raft labelling

Cells were cultured in eight‐well culture chambers (Sarstedt, Numbrecht, Germany) and pre‐treated for 1 hr with 10 mg/ml of MCD before application of H_2_O_2_ (1 hr) or cytokines (4 hrs) and with continuous treatment by MCD or by Liraglutide. After treatment, cells were washed, fixed and kept at 4°C before labelling with 2 μg/ml of biotinylated subunit B of toxin cholera (Sigma‐Aldrich) for 30 min., washing and labelling with streptavidine‐phycoerythrine (Sigma‐Aldrich) for 30 min. After washing and strip mounting, cells were observed by fluorescent confocal microscopy. Insulin secretion and MP shedding were assessed in harvested supernatant. In some experiments TF activity was measured at the surface of unfixed cells.

### Insulin exocytosis blockage and labelling

Rin‐m5f were cultured on eight‐well culture plates. Tetanus toxin (20 nM) was added during 30 min. in a depolarization medium prepared from RPMI medium, to enable toxin internalization 35. Supernatant was withdrawn, fresh medium added and oxidative or cytokine stress applied during 6 hrs. Cells were washed, fixed and permeabilized using Fix and Perm^®^ and kept at 4°C. After 3 washes, guinea pig anti‐rat insulin antibody (dilution: 1:100; Abcam, Cambridge, UK, 30 min., RT) was applied. Washed cells were incubated with FITC‐goat anti‐Guinea pig IgG (dilution: 1/130; Abcam, 30 min., RT). Control conditions consisted of the labelled unstimulated or stimulated cells incubated with the secondary antibody (data not show). After washing and strip mounting, cells were observed by fluorescent confocal microscopy. The proportion of cells exhibiting normal pattern of exocytosis was counted and expressed as per cents of total cells.

### Blockade of the GLP‐1r

Cells were pre‐treated for 1 hr with 200 nM of the GLP‐1r antagonist, exendin fragment (9‐39) (Sigma‐Aldrich) 36. It was previously verified that 200 nM exendin led to maximal inhibition of insulin secretion by Rin‐m5f after 24 hrs and 48 hrs incubation. The supernatant was withdrawn and exendin (9‐39) continuously applied with either cytokines or H_2_O_2_ in fresh medium during 6 hrs.

### Statistical analysis

Data are expressed as mean ± S.E.M. and analysed using GraphPad Prism5 (GraphPad Software, La Jolla, California, USA)^®^. Statistical analysis between two groups was carried out using unpaired Student's *t*‐test. A *P* < 0.05 was considered significant. Experiments were performed in triplicate.

## Results

### Microparticles released in response to oxidative and cytokine stress carry active TF and liraglutide reduces MP‐prompted TF activity

**Figure 1:**
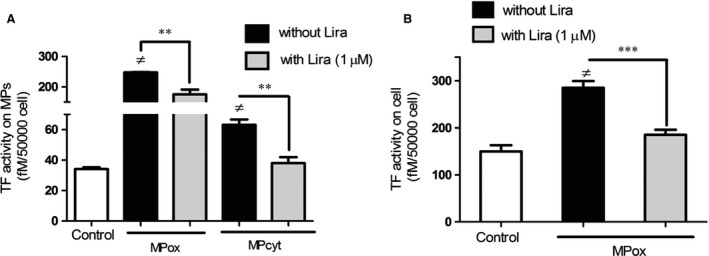
Liraglutide decreases TF activity borne by MPs (**A**) and on target cells (**B**). (**A**) MPs were harvested from supernatants of H_2_O_2_ or cytokine‐treated cells incubated in the presence (grey bars) or not (black bars) of Liraglutide (Lira). The TF activity was assessed by Tenase assay. (**B**) 10 nM MPox were applied to naïve Rin‐m5f cells in the presence or absence of Lira. Empty bars: unstimulated cells. Data normalized as fM TF per 50,000 cells and expressed as mean ± S.E.M. (MPox, MPcyt: MPs produced by H_2_O_2_ or cytokine stimulation; *n* = 6 ≠: *versus* unstimulated cells; **P < 0.01, *P* < 0.0001, ).

Compared to MPs shed from untreated cells, MPs released after oxidative and cytokine treatment bore highly active TF (MPox: 247.7 ± 1.6 fM/50,000 cells and MPcyt: 63.3 ± 5.9 fM/50,000 cells *versus* 37.6 ± 2.4, *P* < 0.0001 and *P* = 0.003 respectively). Incubation of the cells with liraglutide prevented the generation of TF activity in the supernatant with a significant two‐fold decrease in TF activity, regardless of the nature of the stress (MPox: 175.0 ± 15.4 fM/50,000 cells, *P* = 0.01 and MPcyt: 38.0 ± 3.9 fM/50,000 cells, *P* = 0.008, Fig. 1A). Microparticles generated by oxidative stress collected in the supernatant from cells treated by H_2_O_2_ were also able to prompt TF activity at the surface of naïve target cells (from 149.6 ± 13.3 fM/50,000 cells to 285.0 ± 14.3 fM/50,000 cells; *P* < 0.0001) that was reduced in the presence of Liraglutide, TF activity decreasing to 185.1 ± 10.8 fM/50,000 cells (*P* = 0.0001, Fig. 1B).

### Liraglutide prevents the impairment of insulin secretion induced by TF^+^‐MPs

**Figure 2:**
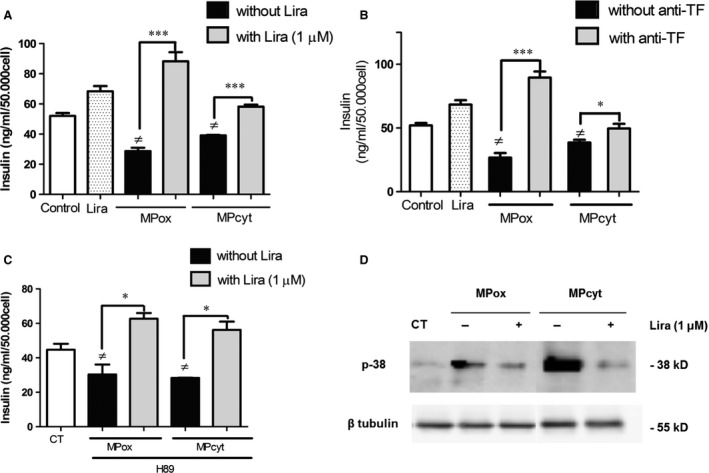
Liraglutide prevents the TF ^+^‐MP‐mediated impairment of insulin secretion (**A** and **B**) through PKA (**C**) and MAP Kinase p38 signalling (**D**). (**A**) Rin‐m5f were incubated with 10 nM MPox or MPcyt in the presence (grey bars) or absence (black bars) of liraglutide (lira) during 24 hrs. (**B**) 10 nM MPs were pre‐treated with anti‐TF (grey bars) or irrelevant antibody (black bars). Secreted insulin was measured in the supernatant. (**C**) Cells were pre‐treated with H89, washed and submitted to 10 nM MPox or MPcyt in the presence or absence of Lira. (**D**) Western blot of MPox‐ and MPcyt‐treated cells lysates. Empty bars: unstimulated cells. Data expressed as mean ± S.E.M. (*n* = 4; ≠: *versus* unstimulated cells; MPox, MPcyt: MP produced by H_2_O_2_ or cytokine stimulation *P < 0.05, ***P < 0.0001).

Microparticles also behaved as cellular modulators of the insulin production, concentrations of insulin being significantly reduced in the supernatants of cells treated by MPox or MPcyt (MPox: 28.8 ± 2.1 ng/ml/50,000 cells, MPcyt: 39.1 ± 0.3 ng/ml/50,000 cells *versus* 52.1 ± 1.8 ng/ml/50,000 cells, *P* < 0.0001 and *P* = 0.02 respectively). Liraglutide counteracted the MP‐driven impairment of insulin secretion and prompted a high yield of insulin secretion similar to that observed in control cells (MPox: 88.3 ± 6.0 ng/ml/50,000 cells, MPcyt: 58.2 ± 1.4 ng/ml/50,000 cells, Fig. 2A). Nevertheless, liraglutide was more efficient in MPox‐treated cells (fourfold yield for MPox, 30% yield for MPcyt).

Pre‐treatment of MPs by an anti‐TF antibody before incubation with target cells prevented the MP‐driven drop in insulin secretion, concentrations in supernatant being significantly increased from 26.8 ± 3.6 ng/ml/50,000 cells to 89.6 ± 4.8 ng/ml/50,000 cells in MPox‐treated cells (*P* < 0.0001) and from 38.6 ± 2.2 ng/ml/50,000 cells to 49.7 ± 3.7 ng/ml/50,000 cells in MPcyt‐treated cells (*P* = 0.02; Fig. 2B). These data indicate a contribution of the active TF borne by MPs to the target cell response. Addition of H89, a PKA inhibitor, to MP‐treated cells led to an approximate 30% decrease in insulin secretion that was completely reversed by liraglutide, regardless of the stress condition (Fig. 2C) suggesting a MP‐driven alteration of the PKA‐dependent response of β cell. Western blots of MP‐targeted cell lysates also indicated an elevated phosphorylation of p38, a MAP Kinase involved in the regulation of insulin secretion and inflammatory MP release, that was also limited by liraglutide (Fig. 2D).

### Liraglutide does not modify the expression of TF at cell membrane

**Figure 3:**
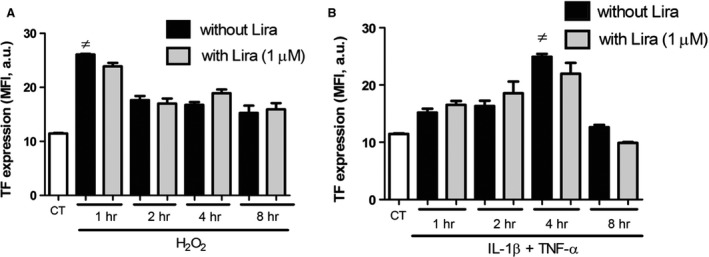
Expression of TF after oxidative (**A**) and cytokine stress (**B**). Oxidative (**A**) and cytokine (**B**) stress, were applied to Rin‐m5f (1–8 hrs). Fluorescence intensity was quantified by flow cytometry after TF‐immunostaining. Data expressed as mean ± S.E.M. (*n* = 3; Lira: Liraglutide; MFI: Mean Fluorescence Intensity; ≠: *versus* unstimulated cells).

Because the enhanced TF activity at cell and MP surface could be the result of an up‐ regulation of TF expression, the kinetics of TF exposure was examined under both stress conditions. After 1 hr oxidative stress, TF expression at cell surface was dramatically elevated (from 11.5 ± 0.2 MFI a.u. in untreated cells to 26.1 ± 0.2 MFI a.u. in 1 hr‐treated cells *P* < 0.0001) and remained significantly higher than baseline thereafter (Fig. 3A). Optimal expression of TF was observed after 4 hr exposure to cytokines (up to 24.5 ± 0.9 MFI a.u. in 4 hr‐treated cells *versus* 11.5 ± 0.2 MFI a.u. in untreated; *P* = 0.001) and TF membrane expression returned to baseline after 8 hrs (Fig. 3B). No effect of liraglutide could be observed whatever the stress (Fig. 3A and B). These data indicate that liraglutide does not modify the expression and exposure of TF, but only alters its activity.

### Raft integrity is targeted by liraglutide in stimulated cells and is critical to MP release, TF activity and insulin secretion

**Figure 4:**
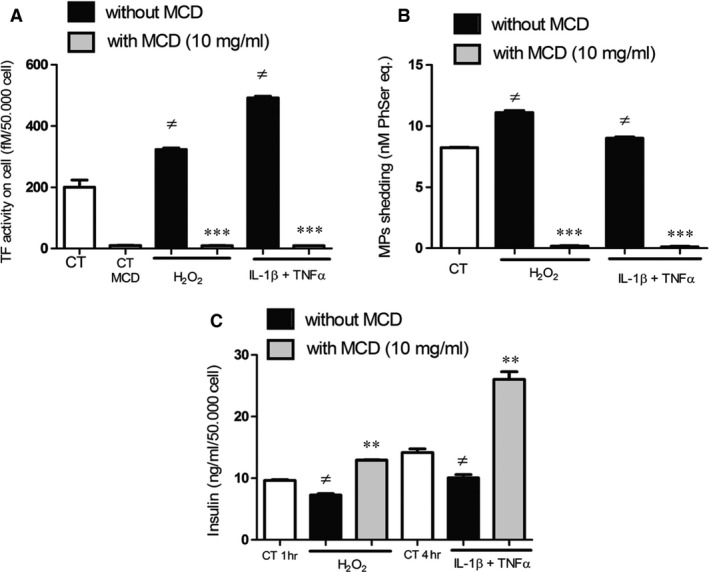
Raft disruption by MCD inhibits TF activity (**A**), MP shedding (**B**) and restores insulin secretion (**C**). Cells were treated by H_2_O_2_ during 1 hr, or cytokines during 4 hrs, in the presence (grey bars) or absence (black bars) of MCD. Empty bars: unstimulated cells, dotted bars: cells treated by MCD alone. Data expressed as mean ± S.E.M. (*n* = 5; MCD: methyl‐β‐cyclodextrin; Lira: Liraglutide; ≠: *versus* unstimulated cells; **P < 0.01, ***P < 0.0001).

**Figure 5:**
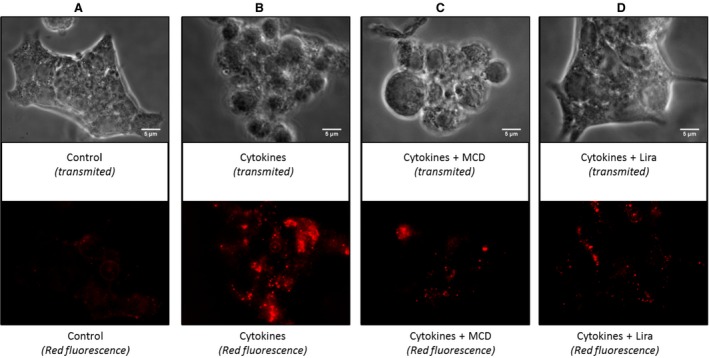
Lipid rafts cluster under inflammatory conditions and are disrupted by liraglutide. After stimulation by cytokines, in the presence (**C**) or absence of MCD (**B**) or in the presence of liraglutide (lira) (**D**), cells were fixed and raft labelled by PE‐cholera toxin. Stimulated and unstimulated (**A**) cells were observed by fluorescent confocal microscopy (×100).

Treatment of Rin‐m5f with MCD completely abolished cellular TF activity (Fig. 4A) and MP shedding under both stress conditions (Fig. 4B). In addition, raft disruption restored insulin secretion (7.2 ± 0.2 ng/ml/50,000 cells in H_2_O_2_‐treated cells *versus* 12.9 ± 0.06 ng/ml/50,000 cells in MCD‐H_2_O_2_‐treated cells, *P* = 0.002; 10.1 ± 0.5 ng/ml/50,000 cells in cytokine‐treated cells *versus* 26.0 ± 1.3 ng/ml/50,000 cells in MCD‐cytokine‐treated cells, *P* = 0.007; Fig. 4C). Staining of the GM1 ganglioside, a marker of lipid rafts, using the fluorescent cholera toxin demonstrated the formation of large lipid raft clusters in response to cytokines by confocal microscopy (Fig. 5B). This major membrane remodelling could not be detected in MCD‐treated stimulated cells in which the raft disruption led to a pattern of small rafts spread over the whole cell surface (Fig. 5C). Raft staining of liraglutide‐treated cells revealed patterns close to those of MCD‐treated stimulated cells (Fig. 5C and D).

### Liraglutide modulates insulin exocytosis and K_ATP_ and Ca^2+^ channels activity

To further investigate the role of raft clustering on insulin secretion and its modulation by liraglutide, ionic channels activity and exocytosis were assessed through pharmacological inhibition and direct staining.

**Figure 6:**
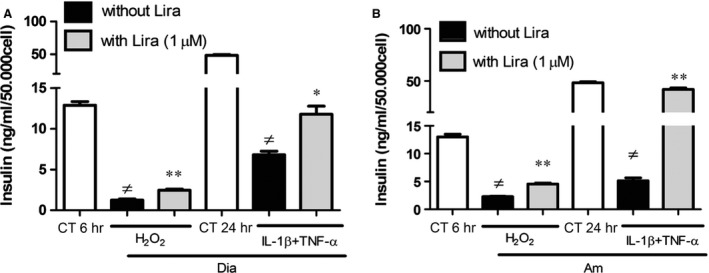
Liraglutide maintains insulin secretion after pharmacological inhibition of K^+^ (**A**) and Ca^2+^ (**B**) channels. Cells were treated with Diazoxide (Dia) (**A**) or Amlodipine (Am) (**B**) and H_2_O_2_ during 6 hrs or cytokines during 24 hrs, in the presence (grey bars) or absence (black bars) of liraglutide (lira). Insulin secretion was assessed by ELISA. Empty bars: unstimulated cells. Data represent the mean ± S.E.M. (*n* = 3; ≠: *versus* unstimulated cells; *P < 0.05, **P < 0.01).

Addition of the K^+^ channel inhibitor, Diazoxide, to H_2_O_2_ or cytokine‐treated cells led to an approximate 85% decrease in insulin secretion that was counteracted by liraglutide. Similar results were obtained with the Ca^2+^ channel inhibitor, Amlodipine (Fig. 6A and B). The alteration of insulin secretion prompted by MPs was also mediated although K_ATP_ and Ca^2+^ channels (data not shown).

**Figure 7:**
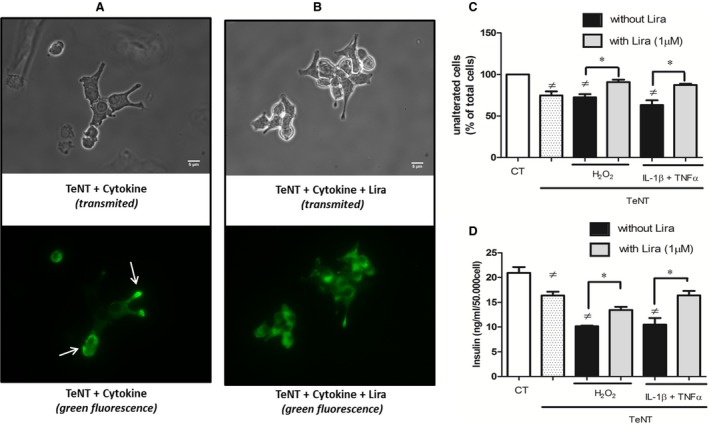
Liraglutide restores insulin release after SNARE blockade by tetanus toxin. Pre‐treated cells with tetanus toxin (TeNT) were incubated with cytokines in the presence (**B**) or absence (**A**) of liraglutide (lira) during 6 hrs. Fixed cells were observed by fluorescent confocal microscopy (×100) after insulin immunostaining. (**C**) Percentage of unalterated cells. (**D**) Insulin released after 6 hrs stress in supernatant. Empty bars: unstimulated cells, dotted bars: cells treated by TeNT alone. Data expressed the mean ± S.E.M. (*n* = 3; ≠: *versus* unstimulated cells; *P < 0.05).

Insulin staining revealed a typical pattern of abnormal exocytosis, the protein accumulating close to the inner leaflet of the membrane and the insulin cytosol content appearing low by comparison. Conversely, treatment by Liraglutide led to a homogenous distribution of the β‐cell insulin content with a decreased proportion of cells that expressed an abnormal exocytosis pattern (Fig. 7A–C). Moreover, 1 μM Liraglutide allowed a higher insulin release in cell supernatant (10.2 ± 0.1 ng/ml/50,000 cells in H_2_O_2_‐treated cells *versus* 13.5 ± 0.6 ng/ml/50,000 cells in liraglutide‐treated counterparts, *P* = 0.04; 10.5 ± 1.3 ng/ml/50,000 cells in cytokine‐treated cells *versus* 16.4 ± 0.9 ng/ml/50,000 cells in liraglutide‐treated counterparts, *P* = 0.03, Fig. 7D).

### The beneficial effects of liraglutide rely on GLP‐1r‐dependent and ‐independent pathways

**Figure 8:**
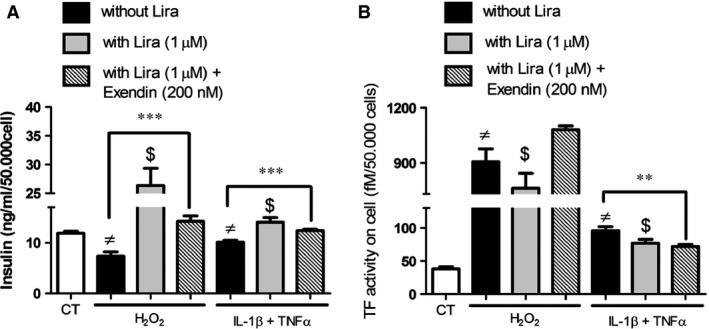
The effects of liraglutide on insulin secretion (**A**) and TF activity (**B**) rely on GLP‐1r ‐dependent and ‐independent pathways. Cells were submitted to 1 hr treatment with the GLP‐1r antagonist exendin (9‐39), before application of H_2_O_2_ or cytokines and exendin during 6 hrs, in the presence (grey bars) or absence (black bars) of liraglutide (lira). Insulin secretion (**A**) and TF activity (**B**) were assessed. Empty bars: unstimulated cells, striped bars: exendin (9‐39). Data expressed as mean ± S.E.M. (*n* = 3; ≠: *versus* unstimulated cells; $: *versus* H_2_O_2_‐ or cytokine‐treated cells; **P < 0.01; ***P < 0.0001).

**Figure 9:**
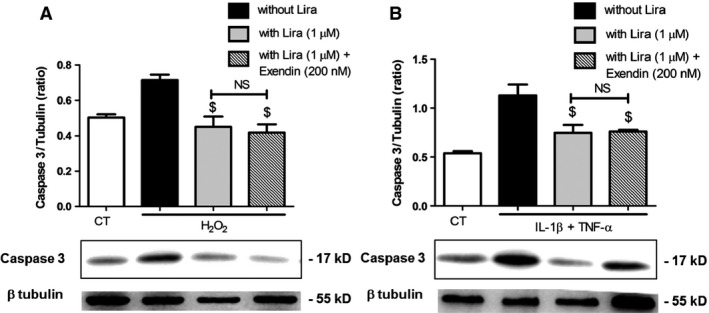
The effects of liraglutide on caspase 3 activation after oxidative (**A**) and cytokine (**B**) stress rely on GLP‐1r ‐dependent and ‐independent pathways. Cells were submitted to 1 hr treatment with the GLP‐1r antagonist exendin (9‐39), before application of H_2_O_2_ or cytokines and exendin (9‐39) during 6 hrs, in the presence (grey bars) or absence (black bars) of liraglutide (lira). Cleaved caspase 3 was demonstrated by Western blot on cell lysates. Empty bars: unstimulated cells, striped bars: exendin (9‐39). Data expressed as mean ± S.E.M. (*n* = 3; $: *versus* H_2_O_2_‐ or cytokine‐treated cells; NS: non‐significant).

**Figure 10:**
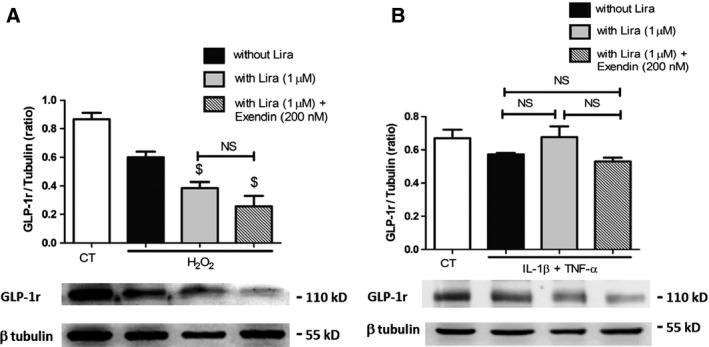
Pharmacological inhibition of GLP‐1r expression in liraglutide‐treated cells after oxidative (**A**) and cytokine (**B**) stress. Cells were submitted to 1 hr treatment with the GLP‐1r antagonist exendin (9‐39), before application of H_2_O_2_ or cytokines and exendin (9‐39) during 6 hrs, in the presence (grey bars) or absence (black bars) of liraglutide (lira). Expression of GLP‐1r was assessed by Western blot in cell lysates. Empty bars: unstimulated cells, striped bars: exendin (9‐39). Data expressed as mean ± S.E.M. (*n* = 3; $: *versus* H_2_O_2_‐ or cytokine‐treated cells; NS: non‐significant).

**Figure 11:**
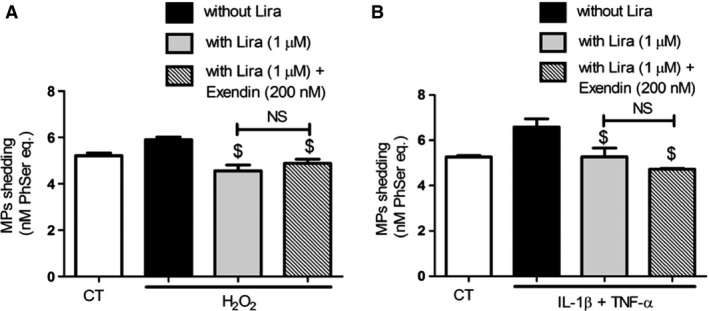
The effects of liraglutide on MP shedding after oxidative (**A**) and cytokine (**B**) stress rely on GLP‐1r ‐dependent and ‐independent pathways. Cells were submitted to 24 hr treatment with H_2_O_2_ or 48 hr treatment with cytokines in the presence (grey bars) or absence (black bars) of liraglutide (lira) and exendin (9‐39). Microparticle concentration was assessed by prothrombinase assay. Empty bars: unstimulated cells, striped bars: exendin (9‐39). Data expressed as mean ± S.E.M. (*n* = 3; $: *versus* H_2_O_2_‐ or cytokine‐treated cells; NS: non‐significant).

Because the effects of liraglutide seemed dependent on membrane remodelling, we suggested that a part of them are independent of GLP‐1r. Cell pre‐treatment by saturating concentration of the GLP‐1r antagonist, Exendin (9‐39), led to a partial abolishment of the liraglutide‐driven insulin secretion, by approximately 50% in H_2_O_2_‐treated cells and 23% in cytokine‐treated cells (Fig. 8A). Interestingly, exendin (9‐39) abolished the liraglutide‐driven reduction of TF activity under oxidative stress, but not under inflammatory conditions with values remaining significantly lower (cytokines: 95.6 ± 6.0 fM/50,000 cells *versus* cytokines‐exendin–liraglutide: 71.8 ± 3.3 fM/50,000 cells, *P* = 0.007; Fig. 8B). Western blots also showed that liraglutide reduced caspase‐3 cleavage by about 30% in H_2_O_2_ or cytokine‐challenged cells, with no significant variation between exendin (9‐39) pre‐treated and untreated cells (Fig. 9A and B). Similarly, liraglutide reduced MP shedding by about 25% in H_2_O_2_ or cytokine‐treated cells, with no significant alteration by exendin (9‐39) pre‐treatment (Fig. 10A and B). No effect on GLP‐1r expression by the exendin‐treated or untreated cells could be observed by Western blot (Fig. 11A and B).

## Discussion

In the present work, we demonstrated that TF borne by MPs modulate insulin secretion in targeted β cells. Because TF activity is highly dependent on membrane remodelling, we questioned the significance of membrane alteration in the cytoprotection exerted by liraglutide 26. Under cytokine and oxidative stress conditions, our data indicate that raft disruption abolishes the raise in TF activity and MP shedding, and restores insulin secretion. Liraglutide treatment led to a disrupted raft‐pattern similar to that observed after MCD treatment. Pharmacological inhibition of raft‐embedded SNARE proteins and Ca^2+^ and K_ATP_ channels showed that liraglutide treatment could maintain insulin secretion. Nevertheless, pre‐treatment at the saturating concentration of exendin (9‐39) before application of liraglutide, did not completely abolish liraglutide effects on TF activity and insulin secretion, whereas caspase‐3 cleavage or MP shedding remained unchanged.

### TF is an early actor in β‐cell dysfunction, whereas MPs maintain durable stress

Because MPs are pathogenic markers of cellular stress that are elevated in T2DM patients, we suggested that β cells are constantly submitted to their deleterious effects. We therefore evaluated MP effects on target cells over one cell cycle duration (24 hrs, see Fig. 2). We indeed identified TF activity and expression as early key players in insulin impairment, time course studies revealing an early cell response (1–8 hrs, see Fig. 3). Therefore, mechanisms of TF‐mediated insulin secretion impairment were assessed after a short time stimulation (from 1 to 6 hrs). Distinct liraglutide modes of action could be observed, thanks to our dual functional and labelling approaches. Indeed, Liraglutide only reduced TF activity, but was ineffective on TF expression at cell membrane, at least during the first 8 hrs of treatment (see Figs 1 and 3 and previous report 26).

### MAP Kinase p38 phosphorylation as a key step in MP‐mediated insulin impairment

Our data are suggestive of multiple pathways targeted by liraglutide in MP‐mediated or direct stress. First, we showed that liraglutide counteracted the p38 MAP Kinase phosphorylation by MPox and MPcyt (see Fig. 2D). On line with our data, p38 was reported to favour endothelial MP shedding in a pro‐inflammatory context 37 and insulin impairment by oxidative stress was associated with increased p38 phosphorylation 38. Moreover, MPs seem to amplify the signal in target cells as MPox and MPcyt mirrored the cell response to the initial stress. Furthermore, the MP deleterious effects could be counteracted by liraglutide, making them a pharmacological target as reported by our team 26. Furthermore, pharmacological inhibition of PKA counteracted the MP‐driven fall of insulin secretion indicating that MP trigger AMP‐dependent pathway (see Fig. 2C).

### Are raft critical for the membrane protein control of insulin secretion?

Apart from signalling pathways, the β‐cell membrane remodelling is another possible target of liraglutide. Indeed, we could evidence that liraglutide altered raft clustering prompted by inflammatory stress (see Fig. 5). These data point at an eventual modulation of the functions of raft‐embedded proteins, among which are the SNARE proteins (syntaxin 1A, SNAP‐25, and VAMP‐2) and voltage‐dependent K^+^ channels (K(V)) 22, 39. Furthermore, insulin secretion was restored by liraglutide when SNARE‐dependent mechanisms were abolished by the tetanus toxin treatment. In view of previous reports showing an enhanced insulin exocytosis after raft disruption 22, it is tempting to conclude that liraglutide maintains exocytosis by acting on raft integrity. This hypothesis is strengthened by our measurement of enhanced insulin secretion after raft disruption by MCD (see Fig. 4C), as reported in INS‐1 and MIN‐6 rat β‐cell lines 22.

Independently of raft distribution, one could consider that Ca^2+^ and K_ATP_ channels, involved in insulin secretion signalling, could constitute a target for liraglutide. We indeed demonstrated that pharmacological inhibition of these two channels led to insulin secretion impairment that was partially counteracted by liraglutide (see Fig. 6). The moderate effect, however, questions an eventual physiological role of rafts. Indeed, other authors have demonstrated that K_ATP_ channels are not embedded in rafts and that Ca^2+^ channels function is not altered by raft disruption 22.

### GLP‐1 analogues exert cytoprotection in ß cells through GLP‐1r ‐dependent and ‐independent pathways

Our data bring new clues to the mode of action of liraglutide as a cytoprotective agent in insulin‐secreting cells challenged by cytokine and oxidative stress. We show that part of the protection exerted by liraglutide is independent of the GLP‐1r, in line with observations in murine endothelium, cardiac and vascular myocytes 40. Indeed, treatment by exendin (9‐39), a GLP‐1r antagonist, counteracted the restoration of insulin secretion and the reduction of TF activity prompted by Liraglutide, but did not significantly alter the other cytoprotective effects triggered by the GLP‐1 analogue, namely MP shedding or caspase‐3 activation (see Figs 9, 10, 11). Because exendin (9‐39) was used at maximal inhibitory concentration and did not significantly modify the expression of the GLP‐1r, it is tempting to consider that liraglutide partly exerts cytoprotection independently of GLP‐1r or through a yet unknown member of the receptor family, as suggested by recent data reported in GLP‐1r knock‐out mice 41. Another possibility would be that the plasma membrane remodelling itself would modulate the potency of the GLP‐1r to activate down‐stream events, as recently reported by Chen *et al*. who demonstrated that specific types of endocannabinoid‐like lipids regulate GLP‐1r signalling 42.

Taken together, our data indicate an integrated β‐cell response to GLP‐1 that combines receptor‐mediated signalling and membrane remodelling. One illustration of such integrated response was given by another team, showing that GLP‐1r activation and raft disruption both lead to an inhibition of raft‐embedded‐K(V) channels, the maintenance of β‐cell excitability and consecutive of insulin secretion 22, 43.

In conclusion, our work demonstrates that TF activity borne by MP released from β cells constitute an amplification loop in the insulin impairment mediated by inflammation. In addition, the ability of liraglutide to limit the raft clustering that promote MP shedding, insulin impairment and TF activity, points to membrane remodelling as a new target in insulin management.

### Study limitation

This study was designed to focus on the β‐cell response to inflammation‐driven membrane response assessed by the shedding of TF^+^‐MPs and raft remodelling. No conclusion can be driven on the integrative response of β cell within the islets architecture, neither on chronic hyperglycaemia‐associated stress and inflammation in the state of diabetes that would require further studies on cultured islets and in animal models of diabetes.

The Rin‐m5f cells were chosen as a model to study the molecular link between prolonged pro‐inflammatory signals and hyperglycaemia on β‐cell functions. These cells are particularly appropriated to the study of β‐cell membrane remodelling in such conditions, but do not respond to short metabolic glucose stimulation. Therefore, confirmation of our data requires studies on primary β cells isolated from islets.

## Conflicts of interest

The authors confirm that there are no conflicts of interest.
